# Vitamin D Supplementation for the Treatment of Depressive Symptoms in Women with Type 2 Diabetes: A Randomized Clinical Trial

**DOI:** 10.1155/2022/4090807

**Published:** 2022-03-03

**Authors:** Sue Penckofer, Monique Ridosh, William Adams, Meghan Grzesiak, Jennifer Woo, Mary Byrn, Joanne Kouba, Patricia Sheean, Colleen Kordish, Ramon Durazo-Arvizu, Diane Wallis, Mary Ann Emanuele, Angelos Halaris

**Affiliations:** ^1^Loyola University Chicago, Health Sciences Campus, Maywood, IL 60153, USA; ^2^Texas Woman's University, Denton, TX 76204, USA; ^3^University of Southern California, Los Angeles, CA 90007, USA

## Abstract

**Aim:**

To determine the efficacy and safety of vitamin D_3_ supplementation in reducing depressive symptoms in women with type 2 diabetes (T2D), depression, and low vitamin D.

**Methods:**

In this double-blind randomized active comparator-controlled trial, women with significant depressive symptoms as assessed by the Center for Epidemiologic Studies Depression (CES-D) scale received weekly oral vitamin D_3_ supplementation (50,000 IU) or an active comparator (5,000 IU) for 6 months. Assessments of vitamin D, 25-hydroxyvitamin D [25 (OH) D], and depression were measured at baseline, 3 months, and 6 months.

**Results:**

A total of 129 women were randomized, from which 119 completed the study (57 in lower dose and 62 in higher dose). Participants had an average 25 (OH) D and HbA1c of 20.8 ng/mL and 7.8%, respectively, at baseline. They were diverse (48% Black) and had a mean age of 50 and T2D for about 8 years. Upon completion of vitamin D_3_ supplementation, serum 25 (OH) D levels increased with 50,000 IU (+34 ng/mL) and 5,000 IU (+10 ng/mL). There was no difference in CES-D scores by treatment dose. Overall, depressive symptoms significantly improved over time with an average CES-D decline of 12.98 points (95% CI: −15.04 to −10.93; *p* < 0.001). Among women with moderate baseline depressive symptoms, those receiving the lower dose had nominally lower depression scores at follow-up than those in the higher dose cohort. Among women with severe baseline depressive symptoms, the improvement in follow-up depression scores was the same regardless of dose.

**Conclusions:**

There was no difference in the dosing effect of vitamin D_3_ supplementation for the treatment of depressive symptoms in women with T2D who present with significant symptoms and low vitamin D. Regardless of the dose, participants' mood improved over time. Further study of vitamin D to target depressive symptoms in comorbid populations is needed.

## 1. Introduction

Depression affects women almost twice as often than men with diabetes [1]. More than 25% of women with diabetes (T2D) have depression and it increases their risk for poor health outcomes [2]. For women having both depression and diabetes, the risk of mortality is significantly higher (relative risk, RR = 3.11) than that for having either diabetes (RR = 1.71) or depression (RR = 1.76) alone [3]. In a recent international study assessing the prevalence and management of depressive disorders in people receiving care in diabetes centers, the female gender was found to be a predictor of major depressive disorder (MDD) [4].

Depression is associated with suboptimal adherence to diet, physical activity, and medications [5]. Antidepressants effectively relieve depression and its related symptoms in persons with T2D [6]. Some antidepressants are twice as likely to assist individuals to achieve glycemic control [7]. A side effect of antidepressants, however, is weight gain which can make compliance challenging [8]. Among persons with diabetes, women are more likely than men to not take their medications because of cost [9]. In addition, although cognitive therapy is an effective treatment for depression and its symptoms in T2D [10], accessibility of trained personnel and insurance coverage for mental health services limit widespread use. Thus, exploration of potential low-cost interventions to treat depression is needed.

Vitamin D supplementation has been suggested as a cost-effective treatment with few side effects for many conditions including depression [11]. Plausible physiologic mechanisms to support the beneficial effect of vitamin D supplementation on depression include its effect on cellular signaling, neurotropic and immunomodulatory processes, and its increased expression of vitamin D receptors in key brain areas [12]. Findings in a recent meta-analysis support that C-reactive protein, an inflammatory marker associated with depression, was reduced following vitamin D supplementation [13].

Earlier systematic reviews and meta-analyses using randomized clinical trials (RCTs) to examine the benefit of vitamin D supplementation on depression have reported mixed results due to study design limitations such as the inclusion of persons without significant depressive symptoms, lack of measurement of vitamin D, and/or inclusion of persons with normal vitamin D levels [14, 15]. One systematic review and meta-analysis reported improvement, but only in those with significant depressive symptoms or depressive disorder [16]. More recent vitamin D supplementation RCTs have found similar mixed results. One meta-analysis reported a favorable improvement in depression ratings (Cohen's *d* = 0.58) following vitamin D supplementation [17] and another concluded that vitamin D supplementation lasting ≥ 8 weeks was most likely to benefit those with MDD [18]. Older adults with significant depressive symptoms enrolled in a recent RCT reported some improvement at 6 months with treatment (55.6% vs. 44.4%, *p* = 0.09) but no difference at 12 months [19]. Another RCT included persons with mild to severe depression and reported no improvement in depression over time; however, one-third of the sample had normal vitamin D levels at baseline and the sample was small [20].

The study is aimed at examining the benefit of vitamin D supplementation using an RCT in women with T2D who have significant depressive symptoms. The use of a non prescription treatment such as vitamin D supplementation for improving depression may benefit women with T2D who have a three-fold greater mortality risk in the presence of both depression and diabetes [3] and a higher rate of depression than men [4]. Further, a prior single-arm trial of vitamin D therapy (50,000 IU per week for six months) improved mood in women with mild to moderate depression living with T2D [21]. Therefore, the current RCT was designed to test whether this higher dose of vitamin D_3_ supplementation (50,000 IU per week) was superior to giving a lower dose (5,000 IU per week) for six months.

## 2. Materials and Methods

### 2.1. Design

This was a randomized, double-blind, active comparator-controlled trial (Clinicaltrials.gov NCT01904032). Women were randomly assigned to a weekly dose of 50,000 IU or 5,000 IU of cholecalciferol (D_3_) using a 1 : 1 allocation for six months. The study was approved as an Investigational Drug Application (IND #126491) through the Food and Drug Administration (FDA) and was approved by the Loyola University Chicago Health Sciences Division Institutional Review Board (IRB). The study was advertised as the Sunshine 2 Study.

### 2.2. Participants

Three major recruitment strategies were used: (1) engaging health centers within a 20-mile radius where an approved flyer was distributed, (2) a waiver of HIPPA authorization to identify potential participants with physicians who agreed to have an informational letter sent, and (3) attending local churches, businesses, and community events to present the study. The inclusion criteria were as follows: (1) female aged 21 and older, (2) having significantly elevated depressive symptoms at screening as measured by a score ≥ 16 on the Center for Epidemiologic Studies Depression Tool (CES-D) or taking an antidepressant medication and having a CES-D score ≥ 12, (3) T2D and under the care of a health care provider, and (4) serum 25-hydroxyvitamin D [25 (OH) D] < 32 ng/mL. The exclusion criteria were as follows: (1) current alcohol or substance abuse disorders, (2) a history of bipolar depression or any other severe or unstable psychiatric disease (e.g., active suicidal ideation), (3) debilitating chronic illness (e.g., cancer and multiple sclerosis), (4) severe complications of diabetes (e.g., blindness or amputation), (5) malabsorption disorders (e.g., Crohn's disease and celiac sprue), (6) elevated serum calcium, (7) taking St. John's Wort unless stopped for 3 weeks prior to enrollment, (8) use of vitamin D supplements (1,000 IU per day or greater) in the past 2 months and unwillingness to discontinue 1 month prior to the study, (9) pregnant, nursing, or planning to become pregnant, and (10) uncontrolled hypertension (systolic > 160 mmHg or diastolic > 100 mmHg). Having active treatment for depression (e.g., antidepressant therapy) was not an exclusion criterion if the person had been under treatment for six weeks or more.

### 2.3. Intervention

#### 2.3.1. Treatment

The vitamin D_3_ supplements (50,000 and 5,000 IU) were provided by Bio-Tech Pharmacal Inc. (http://www.biotechpharmacal.com). The active comparator (lower) dose of vitamin D was based on the Institute of Medicine (IOM) recommendation of 600 IU of vitamin D per day for adults [22]. The weekly 5,000 IU dose approximated that recommendation (i.e., 600 IU × 7 days = 4,200 IU).

#### 2.3.2. Randomization and Blinding

The randomization list was developed by an independent statistician and provided to the research pharmacist who prepared the supplement bottles. A stratified block randomization was used with blocks of random sizes 2, 4, and 6. The two strata were based upon depression symptom severity using the CES-D guidelines [23, 24] and included (1) moderate severity for CES-D scores ≤ 26 and (2) high severity for CES-D scores > 26. It was expected that with the stratified randomization, factors associated with depression would be more evenly distributed. The study supplement bottles were prepared in a blinded fashion so the participant and research team were unaware of the assigned treatment.

### 2.4. Procedures

#### 2.4.1. Phone Screening

Participants who inquired were contacted via phone and the study was described. If interested, age, health information, and questions regarding diabetes and mental health were administered by a nurse to screen for eligibility. The CES-D scale was administered to assess for depressive symptoms. Ineligible participants with significant depressive symptoms were asked to follow-up with their primary healthcare provider.

#### 2.4.2. Study Visits

Eligible participants came to the clinical site. After fasting for 10 hours, participants gave their informed consent. Next, baseline laboratory tests were obtained including venipuncture for serum 25 (OH) D, intact parathyroid hormone (PTH), calcium, and a fingerstick hemoglobin A1c. Vital signs, anthropometric measures (height and weight), and blood pressure were assessed. Subsequently, participants were provided a light breakfast and completed a series of questionnaires assessing their depressive symptoms (CES-D) and other study measures. Finally, the Diagnostic Interview Schedule (DIS), a structured mental health interview that uses the criteria specified in the Diagnostic and Statistical Manual of Mental Disorders IV (DSM-IV) to generate diagnoses for clinical research, was administered by a trained researcher [25, 26]. The DIS was used to obtain the depression history and screen for active suicidal ideation which was an exclusion criterion.

Once all eligibility criteria were confirmed, participants returned within about 10 days to start their assigned vitamin supplementation. A study team member reviewed the vitamin D_3_ supplementation instructions verbally and provided written instructions and a phone number for questions or concerns. Follow-up testing at three and six months utilized the same protocol. Finally, women were contacted by phone at two other time points (two months and between four and five months) for assessment of depressive symptoms and adverse events. Free parking and stepped compensation were provided at data collection visits.

#### 2.4.3. Safety

Vitamin D levels were monitored by the study physician if they exceeded 100 ng/mL. The study physician also monitored serum calcium levels that were >10.5 mg/dL as well as all adverse events. If the treatment was stopped, participants were requested to return for follow-up serum 25 (OH) D concentration within one month as the half-life is approximately two weeks. For these participants, serum 25 (OH) D concentration was collected again at the end of the study. A Data Safety Monitoring Board (DSMB) reviewed reportable events submitted to the IRB.

### 2.5. Measures

#### 2.5.1. Laboratory Measures

Blood specimens were collected and sent to Quest Diagnostics, a CLIAA-certified and approved laboratory (http://www.questdiagnostics.com). Serum 25 (OH) D was measured by liquid chromatography/tandem mass spectrometry which is the standard for the measurement of vitamin D and its components. This method provides a total 25 (OH) D which includes 25 (OH) D_2_ and 25 (OH) D_3_. Intact PTH and calcium were measured using an immunoassay for PTH and spectrophotometry for calcium. Hemoglobin A1c (HbA1c) was used to assess glycemic control with the onsite DCA Vantage Analyzer (Siemens Healthcare Diagnostics, Tarrytown, NY) and fasting blood sugar by Quest Diagnostics using spectrophotometry.

#### 2.5.2. Physical Measures

Anthropometric measures were assessed with a Healthometer Professional electronic scale to the nearest 0.1 kg for weight. Height was measured using a wall-mounted stadiometer. The body mass index (BMI) was calculated as the ratio of weight over height squared. Blood pressure was measured in a standardized fashion calibrated with the DINAMAP *ProCare* 100 Series Monitor (GE Medical Systems, Tampa, FL).

### 2.6. Outcome Measure

The CES-D scale was used to assess the severity of depressive symptoms experienced by women over the past few weeks. The CES-D is a 20-item questionnaire that elicits the frequency of depressive symptoms over the past few weeks using the following response pattern: rarely or none of the time, some or a little of the time, occasionally or a moderate amount of time, and most or all of the time. A score of 16 or higher on the CES-D is significant for positive screening of depression [23, 24]. The CES-D has been used in clinical research for persons with T2D to measure depressive symptoms [27]. It has also been used to assess for remission of depression following treatment [28]. The Patient Health Questionnaire-9 (PHQ-9) includes nine questions about “how often you have been bothered by a series of problems” (e.g., feeling down, depressed, or helpless) in the past two weeks (not at all, several days, more than half the days, and nearly every day). A PHQ-9 score ≥ 10 reflects moderate to severe depression [29, 30]. For this study, the PHQ-9 was used to validate the CES-D with a clinically standardized depression measure. Cronbach's alpha reliability was evident at baseline and three and six months (CES-D: 0.83, 0.89, and 0.91; PHQ-9: 0.71, 0.86, and 0.81).

### 2.7. Statistical Methods

#### 2.7.1. Power

For the power analysis, a repeated measures approach which allows comparisons of the group means across time while adjusting for baseline values and accounting for the correlation between baseline and follow-up observations (i.e., two-tailed test with a type I error rate of 0.05) for the outcome of depressive symptoms (CES-D) was used [31, 32]. Based on data from our prior pilot studies, the current study planned to achieve more than 90% power with group sample sizes of 75 (150 total) to detect a mean difference of approximately 5 points on the CES-D scale between the two dosing groups in a repeated measures design with a compound covariance structure when the standard deviation is 10, the correlation between observations on the same subject is *ρ* = 0.6, and the alpha level is 0.05 [21, 28].

#### 2.7.2. Statistical Analyses

Summary frequencies are provided by treatment allocation for all nominal and ordinal baseline characteristics. Summary statistics are reported for participants' baseline mood and laboratory values as mean with standard deviation when the data were normally distributed; otherwise, these data are reported as median with an interquartile range. An intention-to-treat (ITT) analysis was used to determine if there would be a difference between the vitamin D_3_ doses (weekly 50,000 IU vs 5,000 IU) at three and six months for improving depressive symptoms in women with T2D who were depressed. Linear-mixed effects models were used to estimate the mean change in CES-D and PHQ-9 scores as a function of elapsed time since baseline, treatment assignment, and their interaction while adjusting for baseline values. Because participants could contribute multiple scores to the analysis, random intercepts were allowed for each participant to account for their within-subject correlation assuming a completely general (unstructured) covariance structure. In these ITT models, the denominator degrees of freedom were determined using the method of Kenward and Roger [33]. Regarding model fit, linearity and normality were assessed using residual and QQ plots (respectively), while outliers were assessed using box plots. If the interaction term was not statistically significant at an alpha = 0.05 level, it was removed from the model to estimate the average mean difference in performance between the two treatment groups while controlling for elapsed time since baseline.

Exploratory analyses that further stratified the treat-by-time interaction by the baseline mood score were conducted without any formal null hypothesis tests. These unplanned summaries may provide information to support future investigations of higher dose vitamin D therapy in populations living with type 2 diabetes. All analyses were completed using the statistical analysis system (SAS) version 9.4 (Cary, NC).

## 3. Results

### 3.1. Participant Characteristics

**Figure 1:**
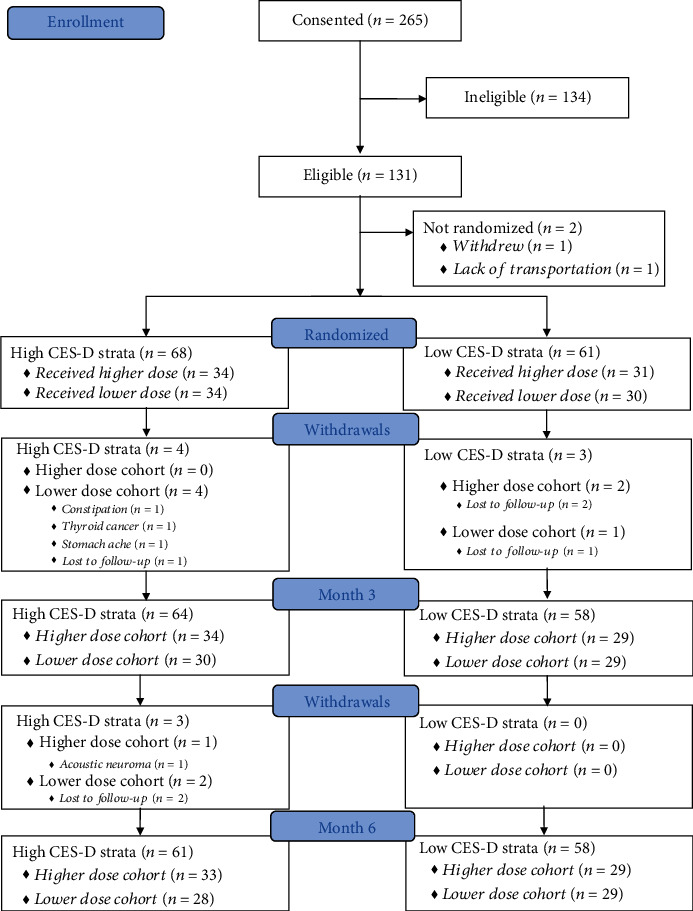
Recruitment and retention flow diagram. Prior to the three-month follow-up visit, four participants in the high CES-D stratum and three participants in the low CES-D stratum discontinued treatment (*n* = 3) or were lost to follow-up (*n* = 4). Similarly, prior to the six-month follow-up visit an additional three participants from the high CES-D stratum discontinued treatment (*n* = 1) or were lost to follow-up (*n* = 2).

Of the 265 individuals consented, 131 were eligible. For those not eligible (*n* = 134), the major reasons were a normal vitamin D level or not being depressed. The goal was to accrue 150 women with complete data into the trial, but the trial stopped for feasibility after consenting 131 participants. Of the eligible individuals (Figure 1), two did not return following their baseline visits and were not randomized. Thus, 129 women were stratified by the severity of their baseline CES-D score and randomized to receive either a higher dose of vitamin D_3_ (*n* = 65) or lower dose of vitamin D_3_ (*n* = 64). Of the 129 baseline participants, 122 (95%) returned for the three-month follow-up visit and 119 (92%) returned for the final six-month follow-up visit.

Baseline characteristics were comparable by treatment allocation, reflecting effective randomization (Table 1). Overall, women averaged 51 years old with 49% identifying as White, 48% as Black, and 3% as other races. Eighteen percent of the sample was Hispanic. The median duration of diabetes was 8 years. As expected, enrollment was highest in the winter and spring months. Significant depression at baseline was evident as the average CES-D score was 28 and the average PHQ-9 score was 12. Baseline vitamin D levels averaged 20.8 ng/mL and indicated insufficiency [11]. The baseline HbA1c averaged 7.7% and was in the target range (7 to 8%) for treatment [34]. Finally, there were comparable levels of PTH, calcium, creatinine, blood pressure, body mass index, and fasting glucose between groups at baseline.

### 3.2. Primary Outcome of Depressive Symptoms and Treatment Allocation

**Figure 2:**
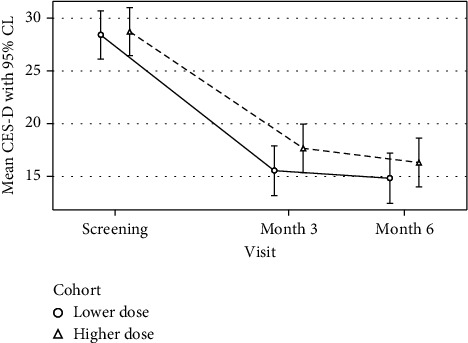
Mean CES-D score by treatment allocation and time. The mean change in CES-D as a function of elapsed time since baseline, treatment assignment, and their interaction from a linear mixed-effects model based on 129 participants contributing 370 CES-D observations.

Figure 2 displays the mean CES-D score by treatment allocation and elapsed time. Controlling for the baseline CES-D score, there was no significant difference in depressive symptoms between participants in the treatment conditions (weekly 50,000 or 5,000 IU) after three months of therapy (*M*_diff_ = 2.08, 95% CI: −1.29 to 5.45; *p* = 0.23) or after six months of therapy (*M*_diff_ = 1.33, 95% CI: −2.06 to 4.72; *p* = 0.44). Both groups exhibited similar and significant improvements in depressive symptoms over time. Controlling for treatment assignment, the average CES-D score declined significantly by −11.94 points (95% CI: −13.97 to −9.90; *p* < 0.001) after three months and by −12.98 points (95% CI: −15.04 to −10.93) after six months of vitamin D_3_ supplementation (*p* < 0.001). Controlling for the baseline CES-D score and treatment assignment, there was no significant change in CES-D scores between three and six months of vitamin D_3_ supplementation (*M*_diff_ = −1.05, 95% CI: −2.13 to 0.03; *p* = 0.06).

Findings were similar using the PHQ-9. Controlling for the baseline PHQ-9 score, there was no significant difference in depression between the two treatments at any time (overall *p* = 0.78). That is, both groups demonstrated similar improvement on the PHQ-9 assessment after three (*M*_diff_ = −5.13, 95% CI: −6.20 to −4.07; *p* < 0.001) and after six months of treatment (*M*_diff_ = −5.61, 95% CI: −6.68 to −4.54; *p* < 0.001). Controlling for the baseline PHQ-9 score and treatment assignment, there was no significant change in PHQ-9 scores between three and six months of vitamin D_3_ supplementation (*M*_diff_ = −0.46, 95% CI: −1.11 to 0.19; *p* = 0.16).

Depression remission was assessed by looking at the categories of how many individuals remained depressed. After three months of treatment, the nondepression (CES-D < 16) rate was 48% (30/63) and 56% (33/59) for women taking the higher and lower doses, respectively. After six months, these rates were 53% (33/62) and 60% (34/57) for women taking the higher and lower doses, respectively. Overall, there was no significant difference in the depression free rate between the two treatment groups at any time. That is, depression remission improved regardless of dose.

**Figure 3:**
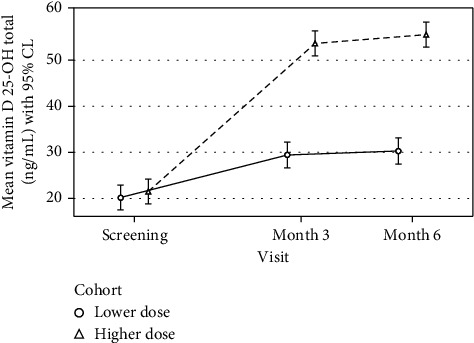
Mean serum vitamin D (25 OH D) by treatment allocation and time. The mean change in the vitamin D level as a function of elapsed time since baseline, treatment assignment, and their interaction from a linear mixed-effects model based on 129 participants contributing 370 vitamin D laboratory measurements.

Participants assigned to 50,000 IU weekly increased their serum 25 (OH) D level by 32.15 ng/mL after three months and 34.06 ng/mL after six months of supplementation. Individuals assigned to 5,000 IU weekly increased their serum 25 (OH) D by 9.23 ng/mL after three months and 10.10 ng/mL after six months. The increases in 25 (OH) D remained steady between three and six months for both groups (higher dose: *M*_diff_ = 1.91 ng/mL and lower dose: *M*_diff_ = 0.87 ng/mL) (Figure 3).

### 3.3. Stratification of Depressive Symptoms and Dosing

**Figure 4:**
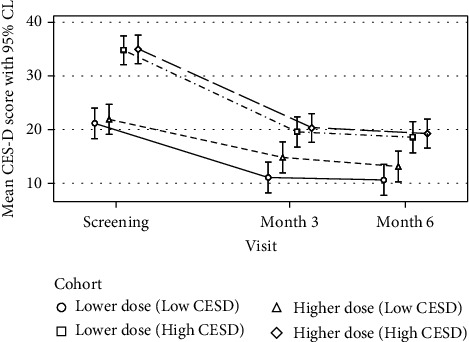
Mean CES-D score with stratification by treatment allocation and time. The mean change in CES-D with stratification as a function of elapsed time since baseline, treatment assignment, and their interaction from a linear mixed-effects model based on 129 participants contributing 370 CES-D observations.

Figure 4 provides exploratory summary data that stratifies the treatment-by-time interaction by baseline CES-D scores. Among participants with less severe baseline depressive symptoms (CES-D ≤ 26), for those taking the lower dose, the mean CES-D score was −3.74 (SE = 2.07) points *lower* after three months and −2.50 (SE = 2.07) points *lower* after six months following vitamin D_3_ supplementation compared to those taking the higher dose. This was not the case for those with more severe baseline depressive symptoms (CES-D > 26 points). The mean CES-D score for those in the lower dose group was comparable to those in the higher dose group after three (*M*_diff_ = −0.73, SE = 1.97) and six months of supplementation (*M*_diff_ = −0.68, SE = 2.02).

### 3.4. Adverse Events of Vitamin D Supplementation

Table 2 provides a summary of the events related to the trial overall. Regarding safety and tolerability, 48 participants experienced at least one adverse event: 21 in the higher dose allocation and 27 in the lower dose allocation. For both groups, the most frequent complaint was increasing depressive symptoms followed by increased systolic blood pressure. Two events thought to be related to the intervention drug were an elevated vitamin D level (>100 ng/mL) and hypercalcemia (>10.5 mg/dL). The elevated vitamin D level was experienced by two participants in the high-dose group as would be expected. Hypercalcemia was noted in 2 participants, one each from the higher-dose and lower-dose groups. In each case, the study drug was discontinued. Follow-up indicated full resolution of elevated lab values by all participants.

## 4. Discussion

The Sunshine 2 Study investigated whether vitamin D supplementation (50,000 vs. 5,000 IU weekly for six months) improved depressive symptoms in women with T2D, significant clinical depression, and lower vitamin D levels. The treatment effect was not different by group, but there was a significant improvement in depressive symptoms over time regardless of the vitamin D_3_ dose.

Empirical evidence of vitamin D supplementation trials has included individuals who either had no or low depressive symptoms and/or did not collect 25 (OH) D levels. Not having baseline criteria for significant depressive symptoms and lower vitamin D levels may have confounded observable benefits. Measurement of vitamin D levels is a strength of the current study. Several meta-analyses of vitamin D supplementation for treatment of depression support the current findings [16, 17]. A recent dose-response meta-analysis reported that a 10 ng/mL increase in 25 (OH) D levels was associated with a 12% decrease in risk of depression [35]. In the current study, an increase in 25 (OH) D of at least 10 ng/mL was observed in both groups with no difference between treatment groups on depression improvement.

There is limited research treating individuals with comorbid conditions such as diabetes and depression. One recent study of women with T2D reported an improvement in mood with weekly vitamin D (50,000 IU) supplementation for 6 months, although it was not an RCT [21]. There have been several small clinical trials. In Tehran, an RCT included individuals with T2D (*n* = 68, 32 per group) who did not have MDD and were not taking antidepressants. They were supplemented with 4000 IU or a placebo daily for 12 weeks and found a significant decrease in depressive symptoms (27.6% vs. 10.8%) compared to placebo (*p* = 0.02) [36]. Another RCT examined women attending a diabetes clinic in Iran who had vitamin D deficiency and received either 50,000 IU vitamin D every 2 weeks (*n* = 26) or a placebo (*n* = 25) for 16 weeks. They reported that anxiety was significantly reduced (*p* = 0.001) and a subgroup analysis noted a decrease in symptoms of depression for those getting the intervention over time (*p* = 0.03), but not for those in the placebo (*p* = 0.11) [37]. The strength of the current study is its larger sample with confirmed depressive symptoms and lower baseline vitamin D.

In our exploratory analyses, there was no difference between dosing and its impact on study outcomes for those with more severe depressive symptoms at baseline. For those with less severe depressive symptoms at baseline, preliminary data suggested that those taking the lower dose of vitamin D of 5,000 IU had a greater decrease in depression scores than weekly dosing of 50,000 IU (*M*_diff_ = −3.74). Further study of vitamin D supplementation and its impact on varying depression severity is needed.

A strength of this study was the effective use of randomization that ensured all measured (and unmeasured) confounders were well balanced as noted in the comparable baseline characteristics of the groups [38]. In addition, having significant depressive symptoms at enrollment was important as prior studies often have not had this as a study inclusion criterion. The stratification by depression severity allowed for exploring potential differences in dosing for those with low and high levels of depression. However, a limitation was that although the 129 women were enrolled and successfully retained, for feasibility reasons, the targeted sample of 150 with power at 0.90 was not achieved. Further, the use of an active-comparator group may obscure whether improvement in depressive symptoms is related to receiving vitamin D therapy. It is possible that the improvement seen in this study was related to other reasons such as participating in the study and having expectations for improvement. However, the increase of vitamin D of 10 ng/mL observed in the current study is similar to a meta-analysis that reported a decrease in the risk of depression with an increase of 10 ng/mL in the vitamin D level [35].

Recent RCTs have not demonstrated the benefit in the use of vitamin D supplementation for the prevention of depression [39] or reduction of episodes of MDD [40]. In a recent US Preventive Services Task Force report, it was noted that populations with specific clinical conditions to evaluate treatment of deficiency for alleviation of their symptoms were not included [41]. Thus, the study of vitamin D for symptom relief in persons with chronic conditions is needed. The findings from the current study demonstrate the benefit in women with T2D who have significant depressive symptomology. Vitamin D_3_ is widely available and well tolerated. Its use in psychiatry is in the preliminary stage of research. Currently, it is reasonable to provide daily vitamin D_3_ of 800 to 1,000 IU to all persons aged 65 and older provided that there are no contraindications and to screen and treat those with depression or cognitive disorders with vitamin D_3_ to a target of >30 ng/mL as an adjunct to usual care [42]. It is without a doubt that depressed individuals will have comorbid conditions such as diabetes, obesity, and cardiovascular disease which will make the study of vitamin D supplementation more complex. Obesity is a risk factor for both diabetes and depression. Given the obesity epidemic, the study of vitamin D deficiency and its relationship to depression, adiposity, and insulin resistance is an important area of exploration [43, 44]. Clinical trials are in progress to allow for a more definitive assessment of the possible antidepressant effects of vitamin D and its impact on the quality of life of these individuals.

## 5. Conclusions

In conclusion, there was no difference in the dosing effect of vitamin D_3_ supplementation for treatment of depressive symptoms in women with T2D who present with significant symptoms and low vitamin D. Regardless of the dose, participants' mood improved over time. Further study of vitamin D to target depressive symptoms in comorbid populations is needed.

## Figures and Tables

**Table 1:** Baseline Characteristics by Treatment Allocation.

Baseline Characteristics	Lower Dose (n = 64)	Higher Dose (n = 65)	Total (N = 129)
Mean age (SD)	51.14 (9.40)	50.02 (12.65)	50.58 (11.13)
Median years with diabetes (IQR)	8 (4-13)	8 (4-12)	8 (4-12)
Race (*N = 128*)			
White	34 (54%)	29 (45%)	63 (49%)
Black	28 (44%)	34 (52%)	62 (48%)
Asian or Pacific Islander	1 (1.6%)	1 (1.5%)	2 (1.6%)
Arabic	0	1 (1.5%)	1 (0.8%)
Hispanic Ethnicity	11 (17%)	12 (18%)	23 (18%)
Use of anti-depressants *(N = 128)*	11 (17%)	12 (19%)	23 (18%)
Season of First Dose *(N = 128*)			
Fall	12 (19%)	15 (23%)	27 (21%)
Winter	22 (34%)	22 (34%)	44 (34%)
Spring	16 (25%)	17 (27%)	33 (26%)
Summer	14 (22%)	10 (16%)	24 (19%)
Screening Mood Measures			
Mean CES-D (SD)	28.41 (8.39)	28.72 (8.50)	28.57 (8.41)
Mean PHQ-9 (SD)	11.91 (4.20)	11.74 (4.29)	11.82 (4.23)
Vitamin D Laboratory Values (ng/mL)			
Median 25 (OH) D_2_ (IQR)	4 (4-4)	4 (4-4)	4 (4-4)
Mean 25 (OH) D_3_ (SD)	18.61 (6.66)	20.63 (6.60)	19.63 (6.68)
Mean Total 25 (OH) D (SD)	20.19 (6.40)	21.49 (6.52)	20.84 (6.47)
Physical and Other Laboratory Measures			
Median PTH (IQR) (pg/mL)	49 (36-69)	49 (38-59)	49 (36-64)
Median calcium (IQR) (mg/dL)	9.4 (9.2 – 9.7)	9.4 (9.2-9.6)	9.4 (9.2-9.6)
Creatinine (SD) (mg/dL)	0.74 (0.14)	0.75 (0.15)	0.75 (0.14)
Mean systolic blood pressure (SD) (mmHg)	128.41 (16.40)	127.43 (14.96)	127.91 (15.63)
Mean diastolic blood pressure (SD) (mmHg)	71.97 (10.64)	70.94 (8.75)	71.45 (9.71)
Mean body mass index (SD)	39.10 (8.28)	37.65 (7.71)	38.37 (8.00)
Mean HbA1c (SD) (%)	7.86 (1.97)	7.68 (1.69)	7.77 (1.83)
Mean fasting glucose (SD) (mg/dL)	170.47 (68.63)	152.52 (48.76)	161.43 (59.90)

*Note*. Valid N = 129 unless otherwise indicated. SD = Standard deviation of the mean. IQR = Interquartile range.

**Table 2:** Summary of Relevant Adverse Events.

Adverse Event	Higher Dose (n = 65)		Lower Dose (n = 64)	
	Number Affected	Number of Events	Number Affected	Number of Events
Passive suicidal ideation	3 (4.6%)	3	1 (1.6%)	1
Significant increase in CES-D (30% increase)	14 (22%)	14	15 (23%)	15
Elevated vitamin D level (≥ 100 ng/mL)	2 (3.1%)	2	0	0
Hypercalcemia (> 10.5 mg/dL)	1 (1.5%)	1	1 (1.6%)	1
Elevated systolic blood pressure (≥ 160mmHg)	6 (9.2%)	8	6 (9.4%)	7
Significant increase in HbA1c (3%)	1 (1.5%)	1	4 (6.3%)	4
Significant increase in creatinine (25%)	1 (1.5%)	1	0	0

*Note.* The number affected may not equal the number of events, principally because an individual can report the same event multiple times.

## Data Availability

The datasets generated during and/or analyzed during the current study are not publicly available but are available from the corresponding author upon reasonable request.
